# Pediatric IBD patients show medication and disease activity dependent changes in NK cell and CD4 memory T cell populations

**DOI:** 10.3389/fped.2023.1123873

**Published:** 2023-06-29

**Authors:** Angeliki Pappa, Julia Mührer, Patricia Gast, Sudheendra Hebbar Subramanyam, Kim Ohl, Moritz Muschaweck, Norbert Wagner, Tobias Wenzl, Klaus Tenbrock

**Affiliations:** ^1^ Department of Pediatrics, University Hospital RWTH Aachen, Aachen, Germany; ^2^ Department of Pediatrics, University Children’s Hospital Zurich, Zurich, Switzerland

**Keywords:** azathioprine, memory T cells, crohn’s disease, ulcerative colitis, natural killer cells

## Abstract

**Objectives:**

CD4+ memory T cells facilitate long-termed adaptive immune responses while NK cells are predominately rapid effector cells with significant functions for both intestinal homeostasis and inflammation. We wanted to study both populations in health and pediatric inflammatory bowel disease (IBD) and correlate them with disease activity and medication.

**Methods:**

We performed flow cytometric analyses of peripheral blood CD4 + CD45RO+ memory T cells and CD3-CD16 + CD56+ NK cells in 30 patients with IBD and 31 age-matched controls and correlated percentages of subsets with disease activity (PUCAI/PCDAI) and medication.

**Results:**

We found a significant reduction of peripheral NK cells in overall IBD patients with both clinical remission and disease activity, which was even more pronounced in patients treated with azathioprine. Otherwise, circulating CD4+ memory T cell populations were significantly enhanced in active IBD compared to controls. Enhancement of memory T cells was particularly found in new onset disease and correlated with disease activity scores.

**Discussion:**

Our single center cohort confirms previous results showing enhanced memory T cell populations in pediatric IBD patients, which correlate with disease activity scores. CD4+ memory T cells are a relevant pathogenic leukocyte population for disease development and perpetuation in IBD. In addition, we found a decrease of NK cells in IBD patients, which was pronounced by use of azathioprine. Surveillance of both cellular populations could possibly serve as biomarker for therapy control in pediatric IBD.

## Introduction

The exact etiology of inflammatory bowel disease (IBD) remains unknown. IBD is suggested to be caused by a dysregulated mucosal immune response to the intestinal microbiota in genetically predisposed hosts (1). IBD are heterogeneous conditions that can be clinically categorized into Crohn's disease (CD) and ulcerative colitis (UC). CD can affect all parts of the gastrointestinal tract and is characterized by granuloma formation and transmural inflammation, while UC predominately localizes to the colon limited to the mucosal layer. Onset of disease starts in about 20% of cases in childhood (2). Pediatric IBD is often aggravated by early onset and severe course of disease (3). T cells possess a dominant role in chronic persistence of IBD, since they significantly contribute to granuloma formation in CD and are highly present within the inflamed mucosa of UC patients (4). Human naïve T cells (CD45RA+) are activated by specific antigen recognition and differentiate towards effector T cells initiating adaptive immune responses. Proportions of effector T cells further develop into memory T cells (*T_m_*). These *T_m_* have enhanced capacities to become activated and produce cytokines upon re-encounter of the specific antigen facilitating a long-termed immunological memory. Up to 60% of lymphocytes residing in the intestinal tissue are CD4+ *T_m_* cells (4). *T_m_* cells can be categorized into circulating (i.e., central memory and effector memory T cells) and non-circulating resident memory T cells [*T_rm_*]. Human *T_m_* are characterized by loss of CD45RA expression and upregulation of CD45RO. Intestinal tissue of IBD patients with active inflammation features enhancement of CD45RO+. Recirculating CD45RO+ *T_m_* cells can be observed in the peripheral blood pool.

Apart from T cells, also cells of the innate immune system like natural killer (NK) cells seem to play a role in IBD. NK cells are lymphocytes with no antigen-specific receptors and are defined amongst others by the expression of CD56 and CD16. NK cells lyse target cells and provide an early source of immunoregulatory cytokines. NK cells have been found to be increased in inflamed mucosa of IBD patients, and NK cell differentiation is also accelerated in the lamina propria, suggesting that NK cells are involved in the disease pathophysiology (5).

## Methods

Patients with UC and CD were diagnosed and treated at the Pediatric Gastroenterology unit of the RWTH Aachen University hospital (Germany). We investigated 30 patients with IBD including 9 patients with UC and 21 patients with CD and 31 age-matched controls and correlated percentages of subsets with disease activity (PUCAI/PCDAI = pediatric ulcerative colitis/ Crohn's disease/ activity indices) and medication (17 female, 13 male, Supplementary Table S1). In some patients we had at least 2, sometimes 3 samples at different time points, which were included in the analysis regarding disease activity and medication. There were no patients with defined underlying genetic disease included in the study. The blood from the control group was obtained from patients that underwent elective surgery e.g., metal removal after fracture several months ago. Mean age was 10.5 in the control group and therefore younger than in the IBD group at tome of analysis (14.5 years), however similar to mean age at diagnosis (11.3 years). The study was approved by the Ethics committee of the RWTH Aachen and written informed consent was obtained from each patient (EK 255/10). One additional 2.5 ml EDTA-sample was obtained during routine blood drawing procedures. We performed a flow cytometric analysis with a focus on CD4 + CD45RO + HLADR+ memory T cells and CD3-CD16 + CD56+ NK cells. Cells were stained in whole blood. Flow cytometric pictures are depicted in Supplementary Figure S1. Antibodies used: CD45-FITC (Invitrogen, Clone HI30), CD45-AmCyan (BD Horizon, Clone HI30), CD3-PB (BD Pharmingen, Clone UCHT1), CD4-APC (Invitrogen, Clone RPA-T4), CD45RO-FITC (Invitrogen, Clone UCHL1), CD16-PE (BD Pharmingen, Clone B73.1), CD56-APC (BD Pharmingen, Clone B159), HLA-DR-PE (Invitrogen, Clone L243). Statistics were done via Graph Pad Prison using one way Anova for multiple comparisons between the different groups and Pearssons correlation.

## Results

### Decreased NK cell expression in patients with CD and patients on treatment with azathioprine

**Figure 1:**
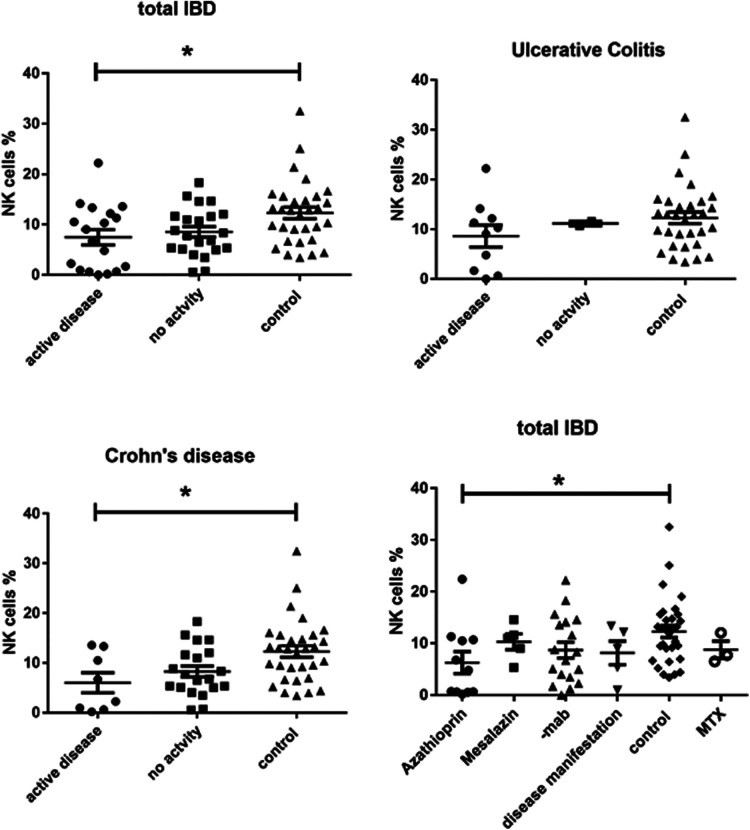
CD3-CD16 + CD56+ NK cell expression in peripheral blood of all active and inactive (PCDAI = 0, PUCAI = 0) IBD patients compared to controls and dissected into the respective disease entities and with regard to medication at timepoint- of analysis, -mab, molecular antibody (Adalimumab or infliximab), MTX, =methotrexate (One way Anova for multiple comparisons, *p < 0.05).

**Figure 2:**
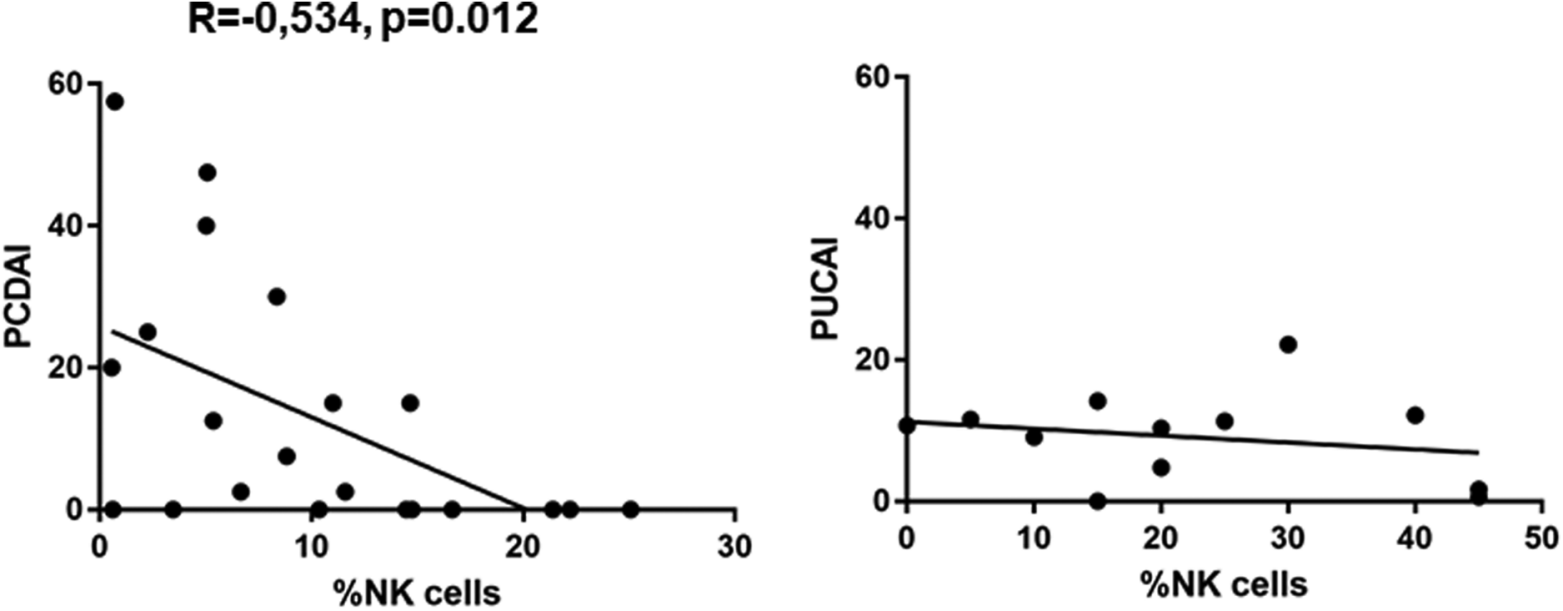
Pearssons correlation of PCDAI and PUCAI with percentages of NK cells (PCDAI *n* = 21, PUCAI *n* = 12).

We found a significantly lower expression of NK cells in Crohn's disease patients with clinical activity (7.472 ± 1.517, *p* = 0.0149) compared to controls (12.29 ± 1.156) (Figure 1). This was not the case in patients with UC. In addition, azathioprine medication resulted in a considerable decrease of NK cell expression (6.29 ± 1.646) vs. controls (12.29 ± 1.156, *p* < 0.05), while all other medications did not affect NK cell expression. PCDAI in CD patients negatively correlated with NK cell expression, while PUCAI did not (Figure 2).

### Enhanced expression of memory T cells in patients with IBD correlates with disease activity

**Figure 3:**
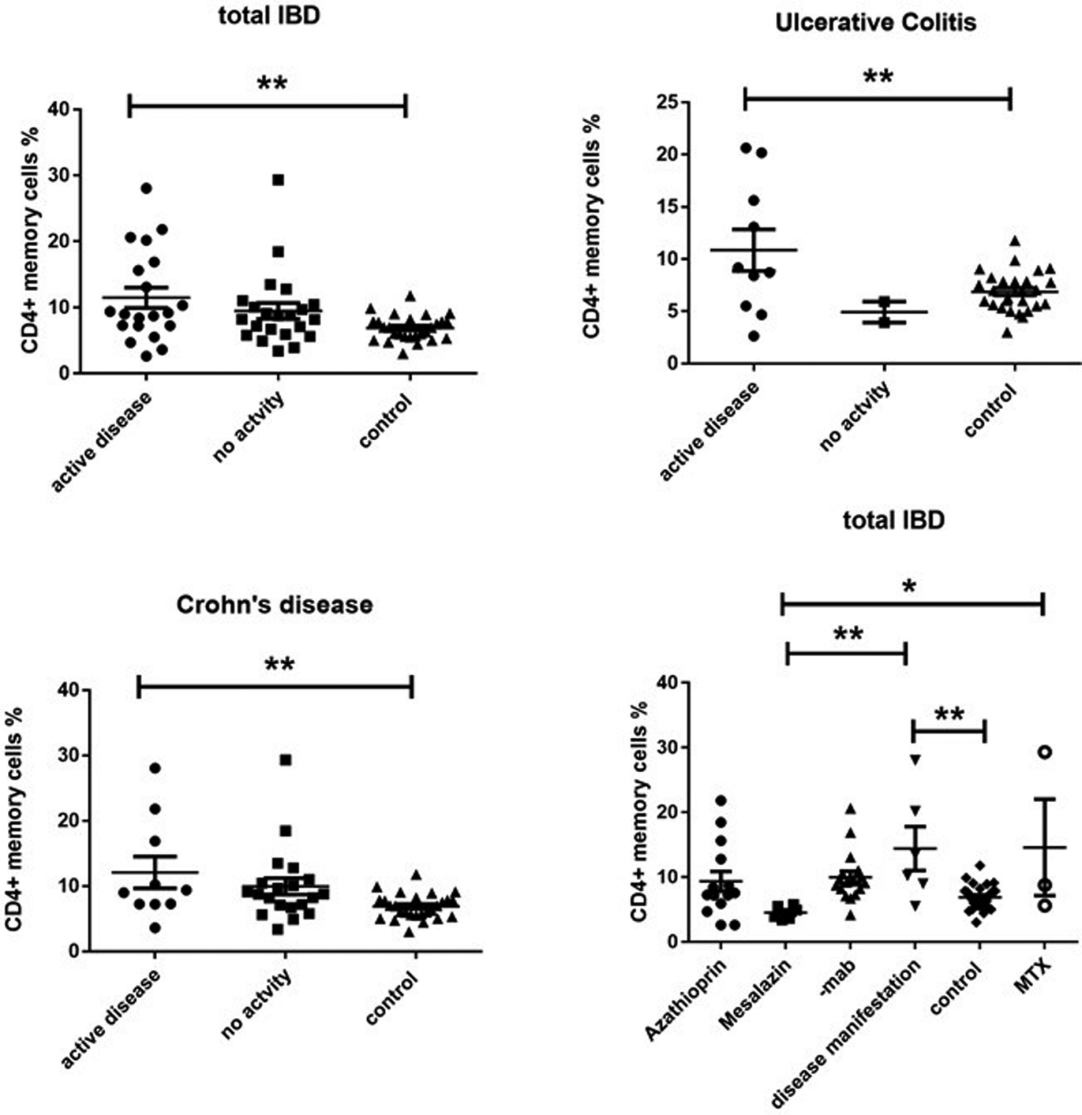
Cd4 + CD45RO + HLA-DR+ memory T cell expression (percentage) in peripheral blood of all active and inactive (PCDAI = 0, PUCAI = 0) IBD patients compared to controls and dissected into the respective disease entities and related to medication at the timepoint- of analysis, -mab, molecular antibody (Adalimumab or infliximab); MTX, methotrexate (One way Anova for multiple comparisons, *p < 0.05, **p < 0.01).

**Figure 4:**
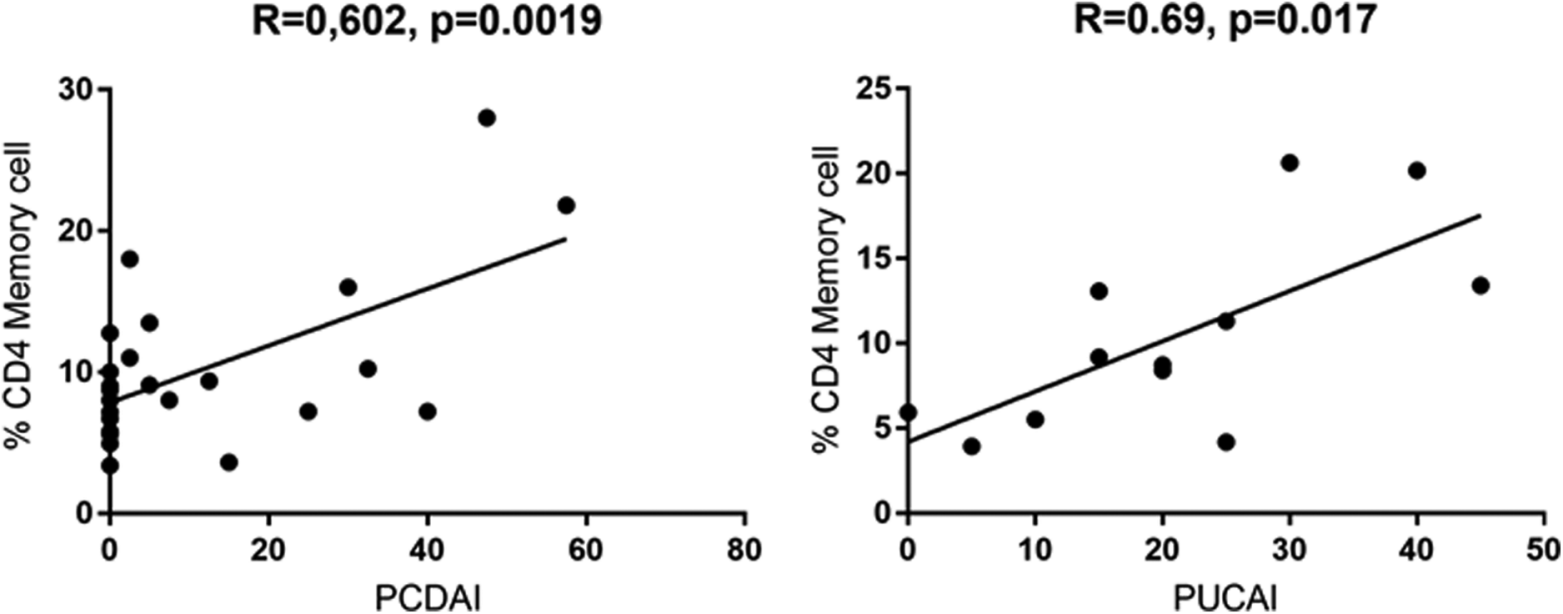
Pearssons correlation of PCDAI and PUCAI with percentages of CD4+ CD45RO + HLA-DR+ memory T cells (PCDAI *n* = 21, PUCAI *n* = 12).

On the other side, CD4 + CD45RO+ memory T cells were significantly enhanced in both active UC (10.77 ± 2.787) and active CD (13.28 ± 2.919) compared to controls (6.870 ± 0.3683), while inactive CD (9.066 ± 1.069) did not show significant differences (Figure 3). Expression of memory T cells was significantly higher at the timepoint- of disease manifestation (14.41 ± 3.399) compared to controls, (6.87 ± 0.36, *p* = 0.006). This difference was not visible anymore in patients that underwent treatment irrespective of the kind. Patients on mesalazine with low disease activity had lower memory cells than the controls (4.525 ± 0.4176, *p* = 0.007) (Figure 2). Enhancement of peripheral CD45RO + HLA-DR+ memory T cells correlated with disease activity scores for both CD and UC (Figure 4).

## Discussion

Our single center IBD cohort study exhibits 2 major findings, which might be of pathophysiological significance. First, CD16+ NK cells are lower in the peripheral blood of pediatric patients with CD. This is in line with a recent publications (6) and in contrast to the enhanced presence of CD16 + NK cells in local inflamed mucosa of the gastrointestinal tract (5). It is currently unclear whether the lower frequencies in the blood are a sign of enhanced migration into the mucosal tissue. In the peripheral blood of patients with IBD, NK cells were shown to produce high amounts of IL-17A and TNF-α ex vivo but had limited killing capability (5). This was accompanied by metabolic alterations including mitochondrial mass and oxidative phosphorylation (7). Azathioprine, which was the only medication that showed a clear decrease of NK cells in our study, preferentially inhibits their proliferation and is able to induce apoptosis at least in resting NK cells (5). This was also shown in a recent publication, in which patients with myasthenia gravis taking azathioprine were investigated (8). In this publication the NK deficiency resulted in decreased IFN-γ production, lower TH1 cells and an increased prevalence of reactivation of endogenous latent herpesviruses. The authors concluded that during the use of azathioprine patients should be monitored for herpes virus reactivation and their findings raise the question of alternative treatment options. In line with that, a NK cell deficiency aggravates experimental colitis in mice (9). On the other hand, a recent paper demonstrated increased numbers of NK cells in newly diagnosed pediatric CD patients compared to healthy controls with enhanced expression of receptors responsible for proper migration into the intestine (10). This is in contrast to our findings, since we found an inverse correlation of NK cells with PCDAI. However, this is merely correlative and dependent on treatment modalities, since we only had the opportunity to measure few newly onset patients. However, a cochrane analysis regarding the use of Azathioprine and 6-mercaptopurine only showed a modest advantage over placebo for induction of remission or clinical improvement in active Crohn's disease in adult patients. They concluded that azathioprine therapy possibly allows patients to reduce steroid consumption and that adverse events are possibly more common in patients receiving antimetabolites compared to those receiving placebo (11). Our data point to the fact that comparable to the data shown in the patients with myasthenia gravis, azathioprine therapy indeed induced NK cell deficiency.

In a recent publication using mass cytometry on colonic samples of adult IBD patients, van Unen et al. showed that innate NK-like CD56+ CD4+ and CD56+ CD8+ T cells were decreased in noninflamed part of the colon compared to inflamed. The reduction of these potential regulatory immune subsets (innate NK-like T cells amongst others) was observed in both CD and UC, and did not associate with disease severity (12).

The other interesting finding is the fact that CD4+ *T_m_* cells are found enhanced in the blood of pediatric patients with IBD. Moreover, we observed clear correlations of peripheral *T_m_* accumulation with disease activity scores and in particular increased frequencies of *T_m_* at disease onset, which decrease irrespective of mode of treatment. Our findings corroborate a paper by Cseh et al. (13), in which they also identified enhanced CD4+ *T_m_* cells in the peripheral blood from pediatric CD patients and is line with several studies in adult patients as well (14, 15). In a recent paper involving a prospective setup in adult patients, a lower CD4 + CD45RO+ cell percentage at the initiation of treatment with TNF-blockers was predictive of a subsequent therapeutic response towards TNF blockers (16) after 12 months. We also performed longitudinal blood analyses in a few patients in our study, but the numbers are far too small to draw any conclusions regarding validity as a potential additive biomarker regarding monitoring of disease activity. These analyses are currently ongoing. Nevertheless, CD4 + CD45RO + HLADR+ memory cells can be easily measured in even small amounts of blood and could therefore be used on a routine basis. Since easy usable biomarkers that predict response to therapy are missing in IBD, any additional marker could be of potential value.

CD4+ T cells represent up to 60% of lymphocytes in the gastrointestinal tract. Most of these T cells are of the CD4+ memory type. Their properties are important for immunity against pathogenic bacteria but need to be tightly controlled to avoid inflammatory responses against harmless commensals. CD4+ *T_m_* can be categorized into effector memory, central memory and tissue resident memory cells. Effector memory and central memory usually recirculate into the blood and they are functionally, transcriptionally and metabolically distinct from tissue resident memory cells. Tissue resident CD4+ T cells are expanded in the mucosa of Crohn's disease patients and more avidly produce IL-17A and TNFα relative to controls (17). Moreover, memory T cells seem to be expanded in CD, but interestingly, healthy siblings of the patients with CD also demonstrated a predominance of memory T cells and elevated expression of β7 integrin already on naïve CD4 T cells compared with controls (18). In murine models it was shown that long-lived colitogenic CD4+ *T_m_* cells residing outside the intestine participate in the perpetuation of chronic colitis (19, 20).

In the same publication mentioned above van Unen et al. identified intestinal HLA-DR + CD38+ effector memory CD4+ T cells to be associated with IBD irrespective of the type. They showed that these cells were be tissue-resident, proliferative, and activated, as assessed at both the protein and RNA level. In addition, they identified an HLA-DR+ EM CD4+ T cell subset in the blood samples that displayed a phenotype similar to one of the most discriminatory cell types in inflamed-IBD biopsies. Notably, a comparison of the frequency of these cells between patients and controls revealed a significantly higher abundance in these patients thus nicely corroborating our data (12).

Due to our staining and gating strategy we cannot define to which subtype the CD4+ *T_m_* belong to. However, it is intriguing that *T_m_* cells measured in blood indeed migrate to and from the gut. The group of Fiona Powrie showed that microbiota-reactive CD4+ T cells are preferably of a *T_m_* phenotype, are present in the peripheral blood as well as in intestinal tissue, and had a diverse T-cell receptor Vβ repertoire, which was identified by TCR sequencing (21). However, TCR repertoire diversities were diminished in patients with IBD (21). The migration of CD4+ T cells into the GI tract is controlled by the α4β7 integrin (22). Circulating CD4+ T cells that express both the α4β7 integrin and the chemokine receptor CCR9 are recruited to the small intestinal lamina propria, while the expression of α4β7 integrin seems to be sufficient for migration into the large intestine (23). The anti-α4β7 integrin antibody Vedolizumab blocks T cell homing to the gut via MAdCAM-1 and thus reduces the recruitment of CD4+ T cell migration and thereby possibly prevents the expansion of new tissue resident memory cells (24). Vedolizumab has been approved for the treatment of UC several years ago (25). Our data point to the fact that *T_m_* cells are of clinical importance, but whether treatment with Vedolizumab changes *T_m_* cell population also in the peripheral blood needs additional studies.

In conclusion, our single center cohort of pediatric patients provides evidence that changes of leukocyte populations in the peripheral blood reflect disease activity in pediatric inflammatory bowel disease. Whether this is of potential value e.g., as easy measurable biomarkers to monitor treatment success needs conformational studies.

## Data Availability

The raw data supporting the conclusions of this article will be made available by the authors, without undue reservation.
