# Supplementary material for: Pediatric IBD patients show medication and disease activity dependent changes in NK cell and CD4 memory T cell populations
Source: Front Pediatr. 2023 Jun 29;11:1123873. doi: 10.3389/fped.2023.1123873 (PMC10345343; doi:10.3389/fped.2023.1123873)
Supplement: Supplementary file 1 [file Datasheet1.pdf]

Supplementary Table 1: summary of the basic characteristics of patients at the timepoint of inclusion into the study (Aza-azathioprine, ADA- adalimumab, 5-ASA- 5 aminosalicylic acid, IFX – infliximab, MTX – methotrexate) and percentages of CD4 memory T cells as well as NK cells

**gender**	**diagnosis**	**age at analysis/ diagnosis in years**	**therapy**	**PCDAI**	**PUCAI**	**Tmem/%**	**NK cells/%**
Male	CD	17/10	ADA, 5-ASA	30		16.8	13.5
Female	CD	16/11	5-ASA, IFX	2.5		11	15.6
Female	CD	14/11	Aza	2.5		18.5	0.7
Female	CD	14/11	IFX	0		9.7	5.8
Male	CD	16/12	IFX	12.5		9.4	2.3
Male	CD	15/5	Aza	0		7.2	0.5
Male	CD	18/15	5-ASA	0		5.8	11
Female	CD	14/12	5-ASA	0		4.9	14.7
Female	CD	17/15	5-ASA	0		3.4	5.4
Male	CD	17/13	IFX	0		8.7	8.8
Female	CD	9/7	Aza, 5-ASA, Budesonid	57.5		21.8	0.6
Male	CD	14/12	5-ASA, IFX	0		10	11.6
Male	CD	17/10	IFX	0		6.7	18.3
Male	CD onset	14/14	none	32.5		10.2	0.9
Male	CD onset	12/12	none	5		13.5	5.4
Female	CD	15/13	MTX	0		5.6	12
Female	CD	18/14	Aza	0		12.8	6.8

Male	CD onset	14/14	5-ASA, prednisone	15		3.6	4.5
Male	CD onset	9/9	none	47.5		28	13.5
Male	CD	10/7	Aza, ADA	40		7.24	0.2
Female	CD	16/12	IFX	5		9.1	4
Female	UC	16/12	5-ASA		5	3.9	11.6
Female	UC	18/14	rectal 5-ASA		10	9	15.9
Female	UC	16/11	ADA		15	13	0
Female	UC	14/11	Aza, 5-ASA, prednisone		20	8.7	10.3
Female	UC	13/12	Vedolizumab, 5-ASA		25	4,2	1.2
Female	UC	11/10	5-ASA, Aza, prednisone		25	4.6	11.3
Male	UC	7/3	IFX, prednisone		30	22	22.3
Female	UC	10/10	none		40	20	12
Female	UC	13/11	Aza, 5-ASA, prednisone		45	2.6	0.6

Supplementary Table 2: summary of the basic characteristics of controls and percentages of CD4 memory T cells as well as NK cells. The percentage of NK cells and CD4 memory cells does not differ between male and female controls.

**gender**	**Age in years**	**Tmem/%**	**NKcells /%**
Male	14	9,05	9,23
Male	13	7,03	12,87
Male	10	7,86	12,83
Female	4	8,93	6,97

Male	4	8,21	6,46
Female	15	5,52	4,43
Female	14	5,00	9,05
Male	7	4,44	16,0
Female	14	4,74	19,05
Female	17	7,85	32,5
Male	3	6,86	12,9
Male	9	6,95	6,65
Female	15	6,00	21,3
Male	3	6,29	14,7
Male	5	9,89	25,0
Male	6	11,7	14,4
Male	16	5,63	16,65
Female	13	9,12	10,35
Male	3	11.5	10.4
Male	10	7,29	13,99
Male	11	5,98	9,55
Female	15	7,52	3,42
Male	11	7,75	9,48
Female	17	5,71	14,31
Male	9	7,84	15,61
Male	16	3.02	13.23
Female	9	6.4	4.5
Male	15	6.99	3.98
Male	12	7,01	7,01
Female	4	5	5,20
Male	17	5.33	9.18

Suppl Fig 1: Gating strategies

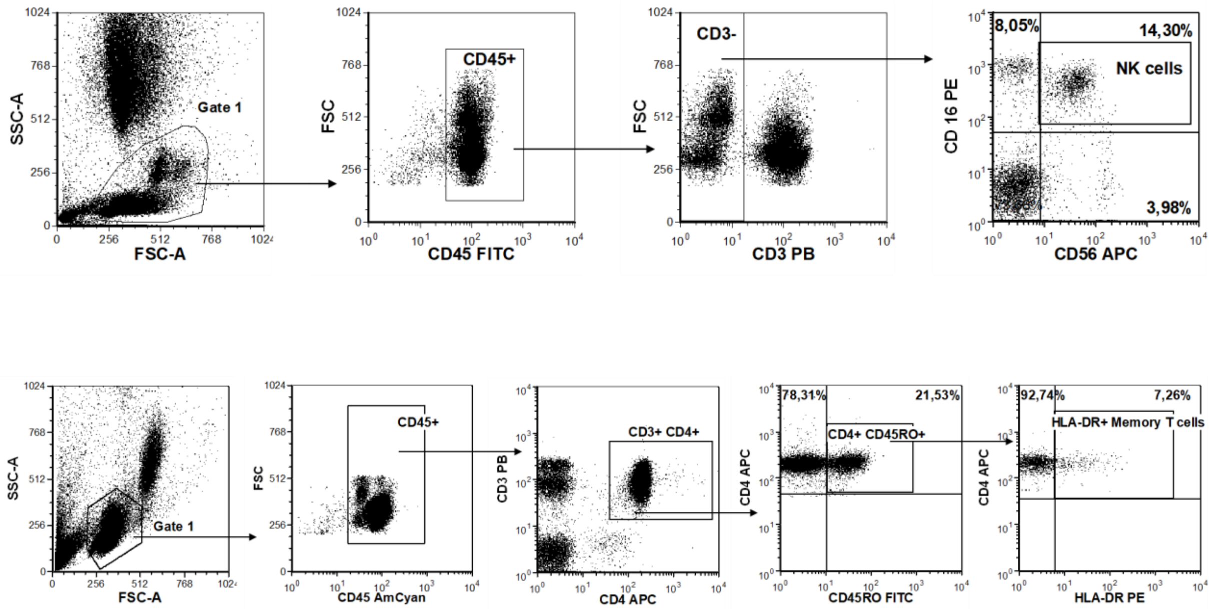

Supp Fig. 1: Gating strategies to determine NK cells and HLA-DR+ Memory T cells in peripheral blood.
